# Neutrophilia with damage to the blood-brain barrier and neurovascular unit following acute lung injury

**DOI:** 10.21203/rs.3.rs-3459515/v1

**Published:** 2023-10-23

**Authors:** Herman Li, Linh Le, Mariah Marrero, Jennifer David-Bercholz, Ana I Caceres, Claire Lim, Wesley Chiang, Ania K Majewska, Niccolò Terrando, Harris A Gelbard

**Affiliations:** a Center for Neurotherapeutics Discovery, University of Rochester Medical Center, Rochester, NY United States; b Department of Neuroscience, University of Rochester Medical Center, Rochester, NY United States; c Department of Neurology, University of Rochester Medical Center, Rochester, NY United States; d Department of Anesthesiology, Duke University Medical Center, Durham, NC, United States; e Department of Biochemistry and Biophysics, University of Rochester Medical Center, Rochester, NY United States; f Department of Cell Biology, Duke University Medical Center, Durham, NC, United States; g Department of Immunology, Duke University Medical Center, Durham, NC, United States; h Department of Immmunology, Microbiology, and Virology, University of Rochester Medical Center, Rochester, NY United States; i Department of Pediatrics, University of Rochester Medical Center, Rochester, NY United States

**Keywords:** Lung, acute lung injury (ALI), LPS, neutrophilia, TLR4, intranasal, hippocampus, CA1, 2-photon

## Abstract

**Background:**

Links between acute lung injury (ALI), infectious disease, and neurological outcomes have been frequently discussed over the past few years, especially due to the COVID-19 pandemic. Yet, much of the cross-communication between organs, particularly the lung and the brain, has been understudied. Here, we have focused on the role of neutrophils in driving changes to the brain endothelium with ensuing microglial activation and neuronal loss in a model of ALI.

**Methods:**

We have applied a three-dose paradigm of 10μg/40μl intranasal lipopolysaccharide (LPS) to induce neutrophilia accompanied by proteinaceous exudate in bronchoalveolar lavage fluid (BALF) in adult C57BL/6 mice. Brain endothelial markers, microglial activation, and neuronal cytoarchitecture were evaluated 24hr after the last intranasal dose of LPS or saline. C57BL/6-*Ly6g*(tm2621(Cre-tdTomato)Arte (Catchup mice) were used to measure neutrophil and blood-brain barrier permeability following LPS exposure with intravital 2-photon imaging.

**Results:**

Three doses of intranasal LPS induced robust neutrophilia accompanied by proteinaceous exudate in BALF. ALI triggered central nervous system pathology as highlighted by robust activation of the cerebrovascular endothelium (VCAM1, CD31), accumulation of plasma protein (fibrinogen), microglial activation (IBA1, CD68), and decreased expression of proteins associated with postsynaptic terminals (PSD-95) in the hippocampal stratum lacunosum moleculare, a relay station between the entorhinal cortex and CA1 of the hippocampus. 2-photon imaging of Catchup mice revealed neutrophil homing to the cerebral endothelium in the blood-brain barrier and neutrophil extravasation from cerebral vasculature 24hr after the last intranasal treatment.

**Conclusions:**

Overall, these data demonstrate ensuing brain pathology resulting from ALI, highlighting a key role for neutrophils in driving brain endothelial changes and subsequent neuroinflammation. This paradigm may have a considerable translational impact on understanding how infectious disease with ALI can lead to neurodegeneration, particularly in the elderly.

## Background:

Lung inflammation following exposure to endo- or exotoxins from infectious agents is inevitable given the unique architecture of this organ: sponge-like, filled with blood, and with an alveolar epithelial layer 100 times the skin’s surface area [1]. Prototypical instigators of lung inflammation are gram-negative bacteria, which contain lipopolysaccharide (LPS) in their outer membrane and are well-known drivers of the innate immune response [2][3]. Intranasal LPS instillation in mice has been extensively used to study the pathogenesis of acute lung injury (ALI) in pneumonia via engagement of Toll-like receptor (TLR) 4 with an accompanying cytokine storm [4]. The ensuing sequelae of lung-to-systemic microenvironment crosstalk can damage end organs, but lung-to-brain signaling is poorly understood. Patients hospitalized with pneumonia can develop neurological symptoms after the resolution of acute infection [5]. Although the effects of systemic LPS on sickness behavior in animal models are well documented, the mechanisms whereby ALI can elicit neuroinflammation have been understudied, likely because the assumption that the primary driver for neurologic disease is hypoxia-ischemia. Indeed, several reports have demonstrated that injuries events such as mechanical ventilation or pulmonary LPS instillation can activate *c-fos* expression in the cortex, amygdala, and thalamus [1][6]. Thus, ALI induced by LPS (like intraperitoneal LPS) has significant peripheral neuroimmune consequences for central nervous system (CNS) neuroinflammation [7].

Sensory neurons, including vagal afferents, innervate the lungs and regulate CNS inflammation via the cholinergic anti-inflammatory pathway [8]. Cell bodies of the jugular-nodose complex below the skull send fibers to the lungs and are transcriptionally modified following ALI [4]. Thus, this complex may represent a complementary mechanism for lung-to-brain communication in health and disease. We have previously demonstrated that activating the cholinergic anti-inflammatory pathway can improve neuroinflammation and cognitive outcomes following surgery and an LPS challenge, including preventing monocyte trafficking into the CNS [9].

Neutrophils are critical first responders in containing invading pathogens, actively resolving infections, and play a pivotal role early in ALI [10][11]. As consequential as neutrophils are in resolving acute infection, neutrophils also cause unintended damage to cellular bystanders in ALI [12]. During ALI, neutrophils are actively recruited to and remain in the lung parenchyma. Once there, neutrophils degranulate and undergo NETosis, which exacerbates the local inflammatory milieu due to the activation of lung-resident dendritic cells and macrophages associated with ensuing lung injury [13][14]. Indeed, lung injury has been associated with a downstream cytokine storm and neutropenia, which have been shown to damage other end organs [15]. Based on these aggregate data, we further investigated the potential of neutrophils to damage the BBB with concomitant CNS injury.

Here, we report on repeated intranasal (i.n) bacterial LPS, which induces ALI and causes neutrophilia, which damages the BBB through brain endothelial inflammation and leads to hippocampal synaptic damage. Notably, the features of this brain injury are strikingly like a murine tibial fracture/fixation model associated with postoperative delirium (POD) and delirium superimposed on dementia (DSD) [16][17]. Our data suggests that previously understudied lung-to-brain signaling from cellular and humoral sources may significantly impact CNS health after ALI.

## Methods:

### Animals and intranasal LPS instillation

Wild-type male C57Bl/6 were purchased from The Jackson Laboratory (Bar Harbor, ME, USA) and housed at Duke University and the University of Rochester Medical Center (URMC). Ly6g^tm2621(cre)Arte^ (Catch-up) male and female mice (as described in [18]) were bred and maintained by the Gelbard laboratory at URMC. The procedures followed to provide blood and tissues from mice were performed in strict compliance with protocols approved by the Duke University Medical Center and University of Rochester Medical Center, Institutional Animal Care, and Use Committee, under the National Research Council Guide for the Care and Use of Laboratory Animals, 8th edition. Duke University and the University of Rochester Medical Center are AAALAC-accredited institutions. Mice were housed under a 12-hour light/dark cycle with access to water and regular chow ad libitum. Mice received intranasal instillation of LPS (Sigma O111:B4 Cat #) (10μg/40μl in saline as a single dose or 30μg/120μl in saline total split among three doses at 0, 24, and 48 hr) was performed under isoflurane (Patterson Veterinary, Greeley, CO) anesthesia. All mice recovered from intranasal LPS instillation and were included in the study [19].

### Collection and analysis of bronchoalveolar lavage fluid (BALF)

Bronchoalveolar lavage (BAL) was performed by cannulation of the trachea and gentle instillation/aspiration (3 times) of 1.0 ml of PBS with protease inhibitor cocktail tablets (Roche, Indianapolis, IN). The lavage fluid was centrifuged at 4000 rpm for 5 minutes, and the supernatant was stored at −80°C for later protein content assessment. The cell pellet was treated with red-blood-cell lysing buffer (BD Biosciences, San Diego, CA), washed, and resuspended in 200 μl of PBS. Total cell counts were determined with a hematology analyzer (Scil Vet ABC, Gurnee, IL), centrifugated onto cytoslides (Cytospin 3, Shandon Inc, Pittsburg, PA) and stained with Diff-Quick (Dade-Behring Inc., Newark, DE). Differential cell counts were obtained by microscopic counting of a minimum of 200 cells/slide, using standard morphological and staining criteria. The total protein in the BALF was measured with a Qubit 3.0 Fluorometer (Invitrogen, Waltham, MA).

### Immunohistochemistry

Mice were deeply anesthetized with isoflurane and transcardially perfused with PBS and 4% PFA. Brains were carefully extracted and post-fixed for 24 hr in 4% PFA. Free-floating coronal brain sections (40μm thickness) were cut using a vibratome and stored in cryoprotectant (30% PEG300, 30% glycerol, 20% 0.1 M phosphate buffer, and 20% ddH2O) at −20° C. Cryopreserved sections were washed three times in 1X PBS followed by another wash in 0.1 M glycine in 1X PBS (to reduce autofluorescence). Sections were subsequently incubated in blocking buffer (1.5% BSA, 3% normal goat serum, 0.5% Triton-X, and 1.8% NaCl in 1X PBS) containing primary antibodies (VCAM1 Millipore Sigma MAB1398Z 1:200; Iba1 Wako PTR2404 1:1000; CD68 Serotec MCA1957GA 1:1000; Homer1 SySy 160006 1:500; PSD95 NeuroMab 75-028 1:500; CD31 Millipore Sigma MAB1398Z 1:250; Fibrinogen Dako A0080 1:200 and fluorescent LPS [20] in blocking buffer (1.5% BSA, 3% normal goat serum, 0.5% Triton-X, and 1.8% NaCl in 1X PBS) overnight at room temperature. Sections were washed thrice in 1X PBS containing 1.8% NaCl before incubating in Alexa Fluor conjugated secondary antibodies (ThermoFisher), again overnight. Finally, sections were washed three times with 1X PBS+1.8% NaCl, mounted on glass slides with Prolong Diamond Antifade Reagent (Invitrogen P36961). When used, the UV-excitable Alexa-633 Hydrazide (Thermo Fisher A30634; 1:1000) was diluted in 1X PBS+1.8% NaCl and applied to sections for 10 min following the first two post-secondary washes; an additional two washes (1X PBS+1.8% NaCl) were performed to rinse the sections of excess Amylo-Glo dye or Alexa-633 Hydrazide.

### Grid pseudo-confocal microscopy and image analysis

Slides were coded throughout imaging and analyses to reduce experimenter bias. We used an Olympus BX-51 microscope equipped with Quioptic Optigrid hardware (for optical sectioning) and a Hamamatsu ORCA-ER camera. Images were acquired using Volocity 3DM software (Quorum Technologies). Within each set, light intensity and exposure settings were kept constant. For ICC, three images were acquired for each experimental replicate. For IHC, six images were acquired for each biological replicate. Synaptic punctae were imaged at 60X magnification (10μm depth, 0.3μm step size). To reduce variability due to differences in vascular coverage (i.e., areas without synaptic punctae), we selected 300 × 300-pixel ROIs from each XYZ stack for analysis. Other markers were imaged at 20X and 40X magnification. Immunostained objects were identified and quantified using custom Volocity workflows. More specifically, for synaptic punctae, the colocalization of spots and objects was used to identify punctae. The “Find Spots” function was used to identify individual punctae based on local intensity maxima, and the “Find Objects” function was used to identify punctae with a minimum intensity threshold; all other quantified objects were identified using the “Find Objects” function. Arterial VCAM-1 vessels were defined as VCAM-1 objects touching Alexa-633 objects. Microglial CD68 was quantified as the sum intensity of CD68 objects contained with Iba1-positive objects. Extravascular fibrinogen quantified as fibrinogen object intensity not colocalized with CD31.

### Cranial Window Surgery

Catch-up mice were anesthetized using a fentanyl cocktail (i.p.) during the cranial window implantation surgical procedure. The fentanyl cocktail consisted of fentanyl (0.05 mg kg), midazolam (5.0 mg kg), and dexmedetomidine (0.5 mg/kg) in saline. Body temperature was maintained at 37°C with a heating pad, and the animal’s eyes were protected with ophthalmic lubricant ointment. All surgical procedures adhered to the aseptic technique. Cranial window implantation surgeries were performed in the Majewska laboratory as described [21][22]. Mice were fixed in a stereotaxic frame, hair was removed with Nair hair removal cream, the dermis was sterilized with three alternating applications of 70% ethanol and beta-iodine, and the skull was exposed through a scalp incision. A 3-mm biopsy punch (Integra) was then used to create a circular score on the skull over the primary somatosensory cortex (S1). A 0.5-mm drill bit (Fine Science Tools, Foster City, CA) was used to drill through the skull for the craniotomy, tracing the 3-mm score. A 5-mm coverslip attached to a 3-mm coverslip (Warner Instruments) by UV glue (Norland Optical Adhesive, Norland) was then slowly lowered into the craniotomy (3-mm side down). The coverslip was carefully secured with C&B Metabond dental cement (Parkell). A custom head plate produced by the emachine shop (http://www.emachineshop.com) (designs courtesy of the Mriganka Sur laboratory, Massachusetts Institute of Technology) was then secured onto the skull with the same dental cement, the rest of which was used to cover any exposed skull and seal the incision site. Mice were administered slow-release buprenorphine (5 mg per kg subcutaneously for 72 h duration) and carprofen (5 mg/kg, i.p. every 24 h) and monitored for 72 h postoperatively. Mice were allowed to recover for a minimum of 30 days and a maximum of 2 months before imaging to reduce parenchymal inflammation and minimize bone regrowth.

### Two-photon intravital microscopy

An Olympus FVMPE-RS with two lasers was used for intravital imaging (Spectra-Physics MaiTai Ti:Sapphire Laser - modified Fluoview confocal scan head, ×20 lens, 0.95 numerical aperture, Olympus). Two-photon excitation was achieved using 100-fsec laser pulses (80MHz) tuned to 840nm with a power of ~50 mW measured at the sample after the objective lens. For anesthetized imaging sessions, mice were anesthetized with the fentanyl cocktail. Fluorescein-dextran (FITC-Dextran) 2,000kDa (50mg/mL in saline 100uL total volume/mouse) was injected via the tail vein 5 min before intravital imaging to visualize the brain vasculature. During and post-imaging, body temperature was maintained at 37°C with a heating pad, and the animal’s eyes were protected with lubricant ointment. Intravital imaging was performed using 4X digital zoom at 512 × 512-pixel resolution. Stacks containing 41 slices (1 mm step size) were imaged every 90 seconds for 15 min to obtain the XYZT stacks used for analysis.

### Statistics:

One- and two-way analysis of variance (ANOVA) with Holm-Sidak’s or Tukey’s multiple comparison tests were used to analyze the IF data as indicated in each figure legend. All data are presented as mean ± standard error (SEM) with significance at p ≤ 0.05.

## Results

**Figure 1:**
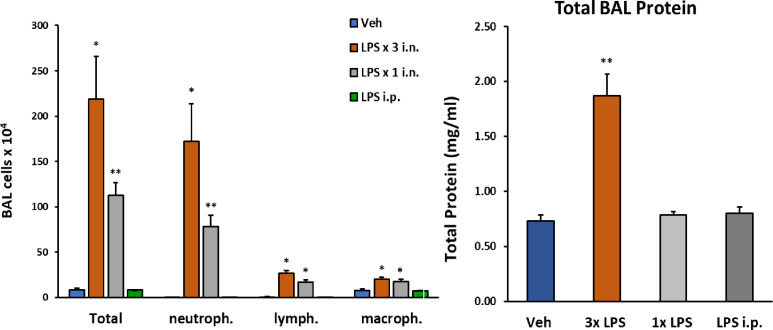
Changes in lung BAL following LPS exposures. *C57/Bl6* male mice (12 weeks old) received either 1 i.n. (10μg/40μl), 3 i.n (30μg/40μl). or 1 i.p. (2mg/kg) administrations of LPS from *Escherichia coli* O111:B4 purified by phenol extraction (Sigma, L2630) in sterile saline (Veh). Mice were sacrificed 24h after last LPS dose (or after single LPS i.n. or i.p). Number of cells detected in BAL fluid was significantly increased following 3 i.n. LPS exposures/daily; total protein content in BAL was also robustly increased following this protocol. Data analyzed with student t-test: p<0.05(*), p<0.01(**), p<0.001(***), p<0.0001(****), data represented as mean (SEM).

We initially determined whether one, two, or three consecutive doses of intranasal LPS would yield a robust inflammatory response in lung airways by analyzing the composition of innate immune cells and proteinaceous exudate in BALF—a single 10μg dose of i.n. LPS increased total cellularity in BALF by 100-fold, while three 30μg consecutive doses of i.n. LPS increased total cellularity by 300-fold (Figure 1).

In contrast, proteinaceous exudate in BALF was significantly increased by over two-fold with our three-dose regimen of LPS, but not after a single exposure to i.n. LPS or for comparison to the effects of systemic inflammation on the lu ng, 2mg/kg i.p. LPS. Neutrophils markedly predominated over lymphocyte and macrophage populations in BALF with both repeated and single exposure to i.n. LPS.

**Figure 2:**
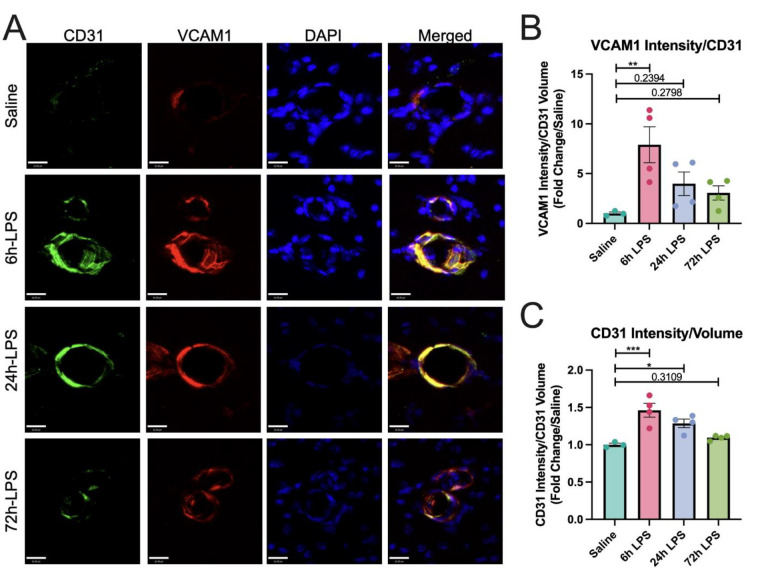
Immunofluorence labeling of VCAM-1 and CD31 expression in hippocampus after intranasal LPS or saline: 12-week-old *C57B/6* mice received intranasal LPS × 3 i.n. and were sacrifice 24h after the last dose of LPS or saline. Brains were removed and processed for IF. Green (CD31^+^), red (VCAM1), blue (DAPI). Scale bar = 16μm (**A**). VCAM1 intensity over CD31^+^ volume quantified (**B**) and CD31^+^ intensity/CD31^+^ volume quantified (**C**). Values presented as mean ± SEM (saline n=3; 6h LPS n=4; 24h LPS n=4; 72h LPS n=4). Analyses were performed as One-way ANOVA with post-hoc analysis: p<0.05(*), p<0.01(**), p<0.001(***), p<0.0001(****).

Having demonstrated that three repeated doses of intranasal LPS could elicit an increased inflammatory response in BALF, we next investigated whether a similar inflammatory response could occur in the brain via activation of peripheral innate immune cells in the lungs, which target brain endothelial cells in the BBB and that can infiltrate CNS parenchyma because of loss of BBB integrity. Thus, we used the same paradigm of intranasal LPS instillation in wild-type C57Bl/6 mice to initially demonstrate that increased immunocytochemical expression of vascular cell adhesion molecule in brain endothelial cells in the BBB of the hippocampal stratum lacunosum moleculare (SLM), which serves as a relay between the entorhinal cortex and CA1 hippocampus [23] [24] (Figure 2).

**Figure 3:**
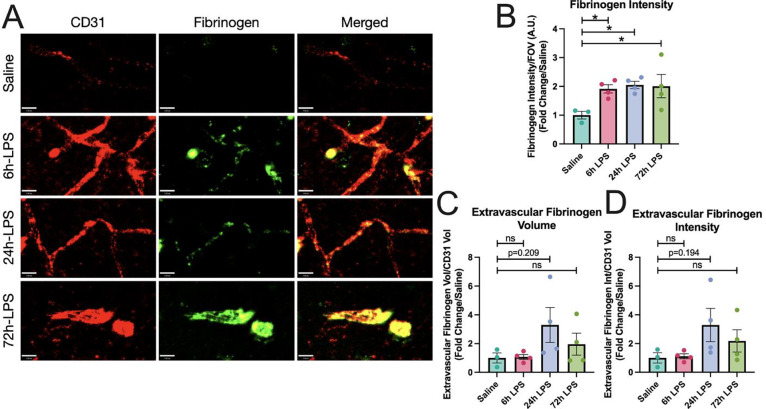
IHC of fibrinogen and CD31 expression in hippocampus after intranasal LPS or saline: 12-week-old C57B/6 mice received intranasal LPS described in Figure 2, followed by sacrifice 24hr after the last dose of LPS or saline. Brains were removed and processed for IF. Red (CD31^+^), Green (Fibrinogen). Scale bar = 16μm (**A**). Fibrinogen intensity over intensity over field-of-view (**B)**, Extravascular fibrinogen volume (**C**) and intensity (**D**) over CD31^+^ volume quantified. Values presented as mean ± SEM (saline n=3; 6h LPS n=4; 24h LPS n=4; 72h LPS n=4). **p<0.01, ****p<0.0001. Analyses were performed as One-way ANOVA with post-hoc analysis: p<0.05(*), p<0.01(**), p<0.001(***), p<0.0001(****).

The effects were statistically robust at 6hr with an 8-fold increase in VCAM-1 normalized to CD31, then diminished over the next two doses. Our results suggest that intranasal LPS administration rapidly promotes a pro-inflammatory cellular environment in BALF that may correlate with platelet and inflammatory leukocyte migration to the BBB in the SLM. While this effect reached a maximum at our 6-hour time point, it remained elevated through the entire period of the three-dose paradigm. Express ion of fibrinogen increased in parallel with CD31 in hippocampal SLM, reflecting paravascular activation, but not a parenchymal deposition, and remained relatively constant after 6, 24, and 72hr dosing of i.n. LPS (Figure 3). 72hr after the third dose of i.n. LPS, fibrinogen, and CD31 showed extensive co-localization.

**Figure 4:**
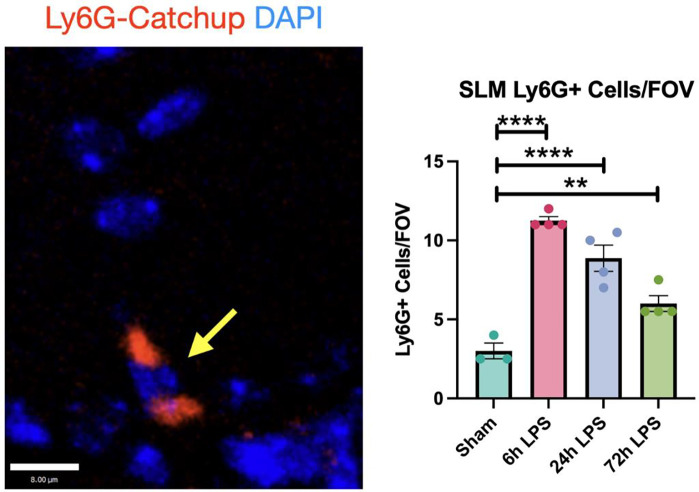
Immunofluorescence of Ly6G+ cells in hippocampus of *Ly6G-Catchup* mice after intranasal LPS or saline: 12 week-old Catchup (C57BL/6-*Ly6g*(tm2621(Cre-tdTomato)Arte)) mice [2] received i.n. LPS, followed by sacrifice 24h after the last dose of LPS or saline. Brains were removed and processed for IF. Red (tdTomato-Ly6G), blue (DAPI). Scale bar = 8μm Values presented as mean ± SEM (sham n=3; 6h LPS n=4; 24h LPS n=4; 72h LPS n=4). **p<0.01, ****p<0.0001. Analyses were performed as One-way ANOVA with post-hoc analysis: p<0.05(*), p<0.01(**), p<0.001(***), p<0.0001(****).

To further determine the role of neutrophil granulocytes in initiating hippocampal injury after exposure to i.n. LPS, we used the Ly6g^tm2621(cre)Arte^ Catch-up murine model [18], which modulates the neutrophil-specific locus, Ly6G, with a knock-in allele expressing Cre recombinase and the fluorescent tdTomato reporter. This model has a very high specificity for neutrophils, with only occasional basophil and eosinophil expression of the fluorophore. In our three-dose paradigm, Ly6G+ cells in the hippocampal SLM increased four-fold after 6hr exposure to LPS relative to the sham control, then decreased to three-fold after the 24hr LPS exposure, and then 2-fold after the 72hr exposure (Figure 4). The diminution in Ly6G+ cellular expression mirrored the relative expression of VCAM-1 and CD31 in wild-type mice (Figure 2).

**Figure 5:**
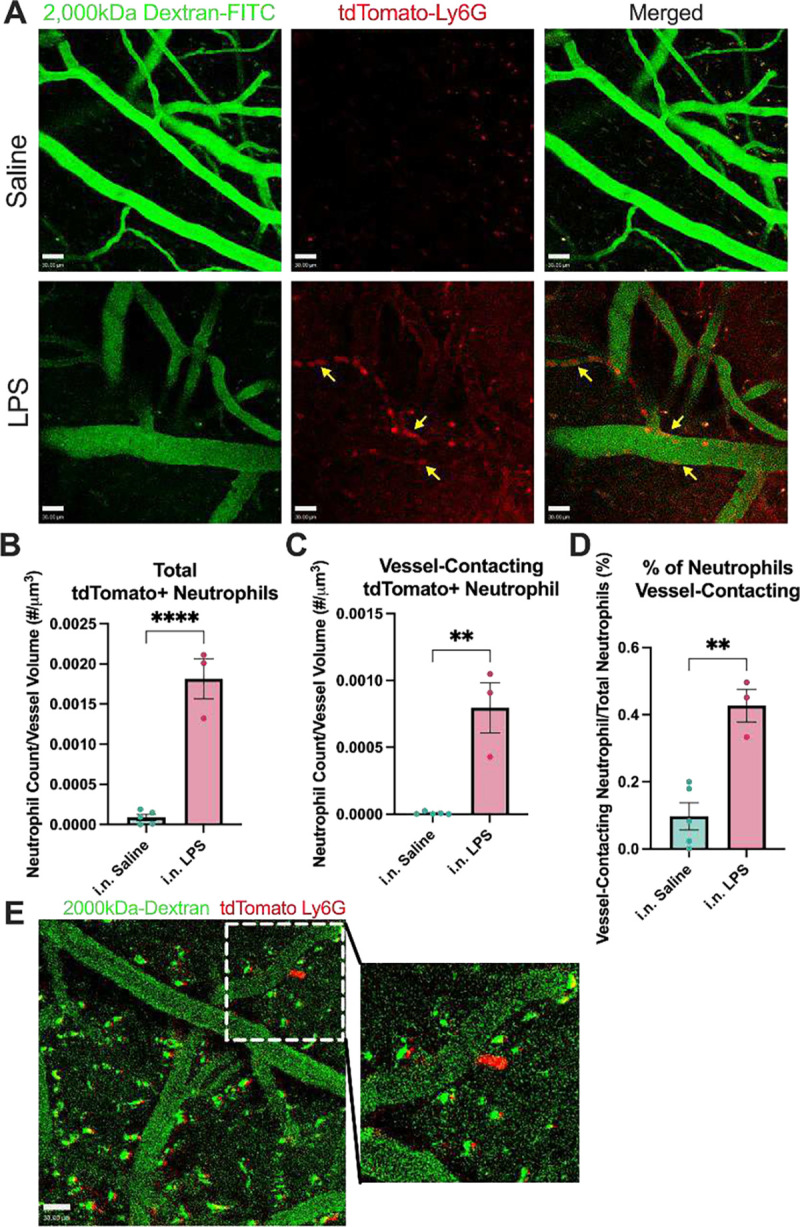
Neutrophils engage along the neurovascular endothelium following ALI. Intravital imaging (2-photon) analysis of fluorescently labeled neutrophils in the somatosensory cortex. 15-week-old Catchup (*C57BL/6-Ly6g*(tm2621(Cre-tdTomato)Arte)) mice were treated with three doses of intranasal (i.n.) LPS (24h apart for 3 days) and were imaged 24hr after the last dose. Z-stack images were taken sequentially every 4 seconds for 100 images (396 seconds). **(A)** Representative images of neutrophil engagement to brain endothelium. Arrows represent neutrophil engagement to adluminal surface of vascular endothelium. **(B)** Quantification of the average number of neutrophils in the field of view per Z-stack image normalized to vascular volume. **(C)** Quantification of the average number of neutrophils contacting the edge of the dextran+ vessels normalized to vascular volume. **(D)** Quantification of the percentage of neutrophils engaging with brain endothelium. **(E)** Representative image of neutrophil extravasation out of brain vasculature into the parenchyma. Green (2000kDa FITC-Dextran), Red (tdTomato-Ly6G). Scale bar = 30μm. Values presented as mean ± SEM (saline n=5; LPS n=3). **p<0.01, ****p<0.0001. Analyses were performed as unpaired t-tests **(B-D)**.

While our postmortem studies investigated the impact of intranasal LPS-induced neutrophilia on the hippocampus, we performed intravital two-photon (2P) microscopy of somatosensory cortical windows in Catchup mice using i.v. 2,000kDa dextran to delineate *in vivo* spatial relationships between neutrophils and endothelial borders in real time, starting 24 hr after the last dose of LPS. 15-week-old Catchup mice underwent open window craniotomies before a 4-week recovery period. Figure 5 demonstrates that neutrophils robustly engage with and migrate through neurovascular endothelium in the somatosensory cortex following ALI. Quantitation of our 2P intravital data (see Table 1) reveals a 38% increase in total neutrophil count per μm^3^, a 112% increase in total neutrophil counts associated with vessels, and a four-fold increase in vessel-associated neutrophils when compared to i.n. Saline. This provides unequivocal evidence for the significance of neutrophilia after ALI and neutrophil interactions with endothelium in the somatosensory BBB. This further suggests that repeated intranasal LPS instillation is sufficient to engage brain endothelium and thereby allow peripheral immune cells infiltration of the brain parenchyma.

**Figure 6:**
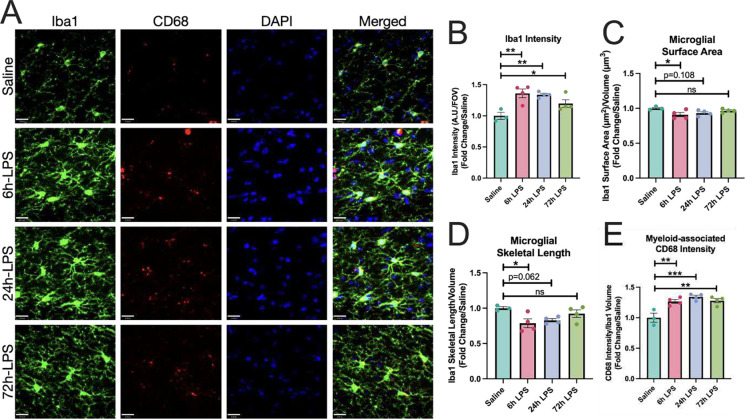
Immunofluorescence of Iba-1 and CD68 expression in hippocampus of C57Bl/6 mice after intranasal LPS or saline. 12-week-old C57B/6 mice received i.n LPS, followed by sacrifice 24h after the last dose of LPS or saline. Brains were removed and processed for IF. Green (Iba1), red (CD68^+^), blue (DAPI) (**A**). Iba1 intensity over field-of-view (**B**), as well as Iba1 surface area (**C**) and skeletal length (**D**) quantified. CD68^+^ intensity over Iba1 volume quantified (**E**) Values presented as mean ± SEM (saline n=3; 6h LPS n=4; 24h LPS n=4; 72h LPS n=4). **p<0.01, ****p<0.0001. Analyses were performed as One-way ANOVA with post-hoc analysis: p<0.05(*), p<0.01(**), p<0.001(***), p<0.0001(****).

We then examined the mononuclear phagocyte markers, Iba-1 and CD68, in hippocampal SLM for changes in expression and morphology during our three-dose i.n. LPS paradigm. Marked increases in Iba-1 expression, with a three-fold increase after the 6hr exposure to i.n. LPS persisted through the 24-hour second dose and diminished to a two-fold increase by the 72-hour exposure (Figure 6). These were accompanied by a sustained increase in expression of the phagocytic marker CD68 [25] normalized to Iba-1 volume. Unsurprisingly, morphologic analyses of mononuclear phagocyte surface area/volume normalized to vehicle control revealed a marked decrease in this ratio at the 6hr LPS exposure time point, as mononuclear phagocytes developed thickened processes and a more amoeboid cell body shape, but over the next few days returned to a more ramified phenotype (Figure 6).

**Figure 7:**
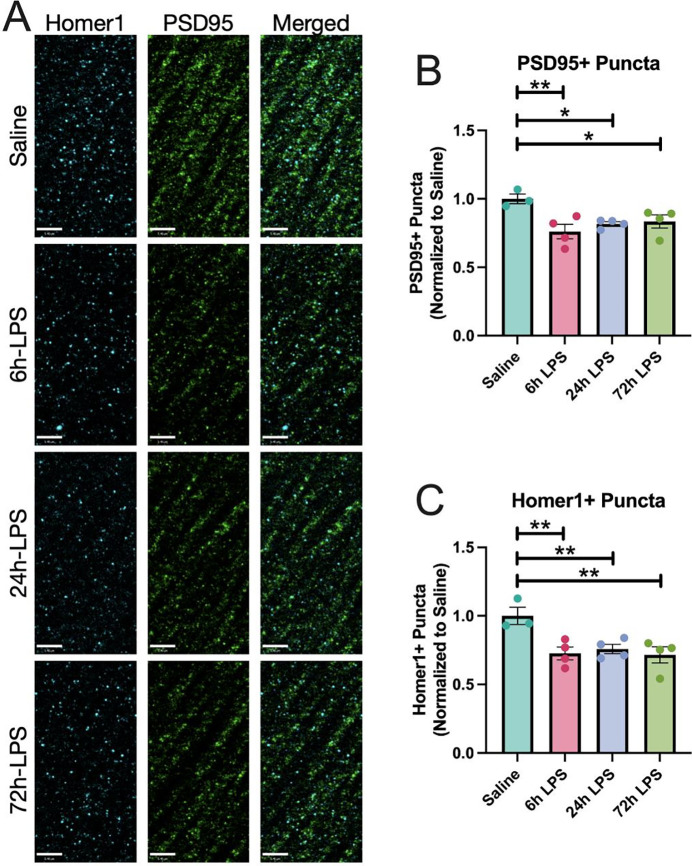
IHC of PSD-95 and Homer 1 expression in hippocampus of C57Bl/6 mice after intranasal LPS or saline: 12-week-old C57B/6 mice received i.n.LPS, followed by sacrifice 24h after the last dose of LPS or saline. Brains were removed and processed for IF. Cyan (Homer1), green (PSD95) (A). PSD95+ (B) and Homer1+ puncta (C) quantified. Values presented as mean ± SEM (sham n=3; 6h LPS n=4; 24h LPS n=4; 72h LPS n=4). **p<0.01, ****p<0.0001. Analyses were performed as One-way ANOVA with post-hoc analysis: p<0.05(*), p<0.01(**), p<0.001(***), p<0.0001(****).

In Figure 7, we demonstrate that repeated intranasal exposure to LPS results in persistent decreased immunocytochemical expression of the post-synaptic scaffold marker PSD-95 [26] and the post-synaptic density glutamatergic marker Homer 1 [27]. Expression of both markers remained with a relatively stable decrease between 24–72 hr.

## Discussion

The results suggest that repeated intranasal instillation of LPS can model a potential route for lung-to-brain neutrophilia with the engagement of brain endothelium, neuroinflammation, and synaptic damage. The pathological changes evoked by intranasal LPS substantiate this method as a model to examine the effects of ALI on blood-brain barrier integrity, which can allow neutrophils to adhere to cerebral endothelium with passage through the BBB into the neurovascular unit (NVU). An unanswered question is whether neutrophil ingress into the NVU in our three-dose paradigm depends on perivascular macrophage or microglial interactions. Without additional studies beyond the scope of the present work, including additional immunohistochemical markers and in situ transcriptomics, a clear distinction between the two cell types cannot be rigorously ascertained [28][29]. Another unanswered question is whether these neutrophils return to peripheral circulation further to escalate neuroimmune crosstalk during ALI [30]. Regardless, in our study, both three-dose ALI and two daily i.p. injections of 1mg/kg of LPS were sufficient to decrease the expression of hippocampal post-synaptic glutamatergic synapses.

Furthermore, do these neutrophils return to peripheral circulation to further escalate neuroimmune crosstalk during ALI [30]? In this study, two daily i.p. injections of 1mg/kg of LPS were sufficient to observe these phenomena.

We have shown that three doses of intranasal LPS significantly increased the neutrophil inflammatory response in BALF over a 72-hour time course. An unanswered question is why there is a temporal dissociation between the cumulative response to 3 doses of i.n. LPS in the lung versus activation of cerebral endothelial inflammation in the hippocampus, which reaches a maximum at 6hr and then decreases between 24–72hr. Despite the time course of these phenomena, activation of mononuclear phagocytes assessed by immunohistochemical markers with a phagocytic phenotype and a decrease in expression of post-synaptic markers endures between 6–72hr. This, in turn, suggests that maximal cerebral endothelial inflammation may be transient but triggers a subacute enduring effect in microglial activation with post-synaptic pruning. Future experiments with behavioral endpoints focusing on indices of delirium and memory impairment will be necessary to assess the functional correlates of BBB inflammation in hippocampal SLM. Interestingly, we demonstrated that in our paradigm of ALI, there was little to no evidence of the direct influence of data with radiolabeled LPS injected by either an intraperitoneal or intrajugular route [32][33]. This is likely explained by the downstream effects of peripheral immune innate cells that activate brain endothelial cells to secrete pro-inflammatory cytokines. While the direct molecular mechanisms of this communication to microvascular endothelial cells are still being investigated in our paradigm, in vitro evidence suggests that pro-inflammatory cytokines such as IL-1β and TNFα alter transmembrane resistance (a measure of integrity) and upregulate endothelial adhesion molecules such as VCAM-1 [34].

## Conclusion:

In the model we employ here, pathologic changes include ALI, activation of endothelium adhesion markers in the hippocampus, paravascular injury from dysregulation of fibrinogen, neutrophil contact with cerebral endothelium and ingress into CNS parenchyma and persistent diminution of post-synaptic markers. While many questions beyond the scope of the present work remain unanswered, future studies should address the following: (1) does CNS neutrophilia from ALI lead to NETosis? If so, what are the downstream consequences? (2) why does the increase in lung pathology after repeated LPS-induced ALI not correlate with the time course of cerebral endothelial inflammation? (3) what are the behavioral consequences of this type of CNS injury, particularly in the aging brain? (4) does aberrant vagal neurotransmission from the jugular-nodose complex instigate BBB inflammation with subsequent downstream effects on synaptic pruning, or are these due to cellular and humoral causes? Further work is needed in identifying the question above. Our data underscore the importance of lung-brain neuroimmune signaling pathways and their applicability to CNS disease.

## Figures and Tables

**Table 1:** Quantification of 2P intravital data. Intravital imaging (2-photon) quantification of fluorescently labeled neutrophils in the somatosensory cortex. 15-week-old Catchup (*C57BL/6-Ly6g*(*tm2621*(*Cre-tdTomato)Arte*)) mice were treated with three doses of intranasal (i.n.) LPS (24h apart for three days) were imaged 24 hr after the last dose. Z-stack images were taken sequentially every 4 seconds for 100 images (396 seconds).

Total Neutrophil Count (# Neutrophils/um^3^):
i.n. Saline: 8.790×10^−5^ ± 3.721×10^−5^
i.n. LPS: 1.814×10^−3^ ± 2.478×10^−4^

Vessel-associated Neutrophil Count (# Neutrophils/um^3^):
i.n. Saline: 7.079×10^−6^ ± 4.605×10^−6^
i.n. LPS: 7.951×10^−4^ ± 1.879×10^−4^

Percent of total neutrophils vessel-associated (%):
i.n. Saline: 0.09715 ± 0.04025
i.n. LPS: 0.4270 ± 0.04681

## Data Availability

All data generated or analyzed during this study are included in this published article [and its supplementary information files].
